# The evidence based dilemma of intraperitoneal drainage for pancreatic resection – a systematic review and meta-analysis

**DOI:** 10.1186/1471-2482-14-76

**Published:** 2014-10-08

**Authors:** Ulrich Nitsche, Tara C Müller, Christoph Späth, Lynne Cresswell, Dirk Wilhelm, Helmut Friess, Christoph W Michalski, Jörg Kleeff

**Affiliations:** 1 Department of Surgery, Klinikum rechts der Isar, Technische Universität München, Ismaninger Strasse 22, 81675 Munich, Germany; 2 Institute of Medical Statistics and Epidemiology, Klinikum rechts der Isar, Technische Universität München, Munich, Germany; 3 Division of Surgical Oncology, Department of Surgery, Oregon Health and Science University, Portland, OR, USA

**Keywords:** Pancreas, Pancreatic, Resection, Drainage, Drain, Intraperitoneal

## Abstract

**Background:**

Routine placement of intraperitoneal drains has been shown to be ineffective or potentially harmful in various abdominal surgical procedures. Studies assessing risks and benefits of abdominal drains for pancreatic resections have demonstrated inconsistent results. We thus performed a systematic review of the literature and meta-analyzed outcomes of pancreatic resections with and without intraoperative placement of drains.

**Methods:**

A database search according to the PRISMA guidelines was performed for studies on pancreatic resection with and without intraperitoneal drainage. The subgroup ‘pancreaticoduodenectomy’ was analyzed separately. The quality of studies was assessed using the MINORS and STROBE criteria. Pooled estimates of morbidity, mortality and length of hospital stay were calculated using random effects models.

**Results:**

Only two randomized trials were identified. Their results were contradictory. We thus included six further, retrospective studies in the meta-analysis. However, with I^2^ = 68% for any kind of complication, the estimate of inter-study heterogeneity was high. While overall morbidity after any kind of pancreatic resection was lower without drains (p = 0.04), there was no significant difference in mortality rates. In contrast, pooled estimates of outcomes after pancreaticoduodenectomy demonstrated no differences in morbidity (p = 0.40) but increased rates of intraabdominal abscesses (p = 0.04) and mortality (p = 0.04) without intraperitoneal drainage.

**Conclusion:**

Although drains are associated with slightly increased morbidity for pancreatic resections, routine omission of drains cannot be advocated, especially after pancreaticoduodenectomy. While selective drainage seems reasonable, further efforts to generate more reliable data are questionable because of the current studies and the presumed small differences in outcomes.

**Trial registration:**

Systematic review registration number CRD42014007497.

## Background

Intraperitoneal drains are frequently placed at the end of complex abdominal operations [1]. These drains were initially thought to enable the early identification of hemorrhage or anastomotic dehiscence, allowing timely re-operations. It has also been hypothesized that prophylactic intraperitoneal drainage can prevent additional interventions for intraabdominal fluid collections [2]. However, many studies, including randomized controlled trials, systematic reviews and meta-analyses have shown that routine drainage after various general surgical procedures such as appendectomy, cholecystectomy, hepatectomy, colectomy and gastrectomy does not reduce the number of complications [1]. Some of these studies even found an increased risk of complications with drains [2,3]. This is thought to be a result of an artificial access to the peritoneal cavity, of an inflammatory response to the drain as a foreign body, and of increased pain due to the drain itself. Despite a high level of evidence favoring the omission of drains, clinical practice has only slowly changed [1].

The evidence for or against intraperitoneal drainage after pancreatic resection is much less clear. Here, drains are placed to detect hemorrhage in the immediate postoperative period, but are also frequently left in place for an extended period of time to allow for detection of leakage of the pancreatic anastomosis/pancreatic stump [2]. Though this concept has not been substantiated by reliable evidence, it is the standard of care all over the world. Interestingly, it had already been challenged in 1992, when Jeekel and co-authors published a series of 22 patients without intraperitoneal drainage [4]. Here, omission of drains was not associated with an increased rate of complications. In 1998, the first retrospective study from the Memorial Sloan Kettering Cancer Center (MSKCC) was published, comparing patients with intraperitoneal drainage to those without. There was “no statistical difference in the rate of fistula, abscess, CT drainage, or length of hospital stay” [5]. Because of these findings, the MSKCC group at that time designed and conducted a randomized controlled trial to generate better evidence. The results were published in the Annals of Surgery in 2001 [6]. 179 patients with pancreaticoduodenectomy or distal pancreatectomy had been randomized to the drain or no drain group. There was no significant difference in the number or type of complications between the groups. Thus, for the first time, level 1b evidence had shown that intraperitoneal drains may not be necessary after pancreatic resection. Several years later, the authors of this randomized study validated their promising initial results by another retrospective report on 1,122 patients of their institution. They again advocated the benefits of omitting drains [7]. However, the latter retrospective non-randomized analysis also revealed that the participating surgeons still decided to place a drain in roughly half of all pancreatic resections.

Subsequently, with only very few exceptions, several retrospective reports did not identify significant differences between drain and no drain groups regarding morbidity, pancreatic fistula, abscess, interventional radiology procedures, re-operation, length of hospital stay, or mortality [5,7-11]. These studies demonstrated that drains could probably be safely omitted after pancreatic resections. However, the available level of evidence was still not convincingly enough to change clinical practice. A main reason for this was that the prospective, randomized trial from the Memorial Sloan Kettering Cancer Center [6] had not reported complications (and particularly, leak rates) according to current standards – owing to the lack of such reporting standards at that time. This reasoning prompted the initiation of a multi-center, randomized trial whose results have recently been reported [12]. In this study, 137 patients with pancreaticoduodenectomy for benign or malignant pancreatic pathologies were randomized to placement or no placement of an intraperitoneal drain. The study was conducted at nine academic high-volume pancreas surgery centers in the United States. Randomization by demographics and clinical characteristics was performed thoroughly, minimizing the risk for any systematic bias. Unexpectedly, the group of patients without a drain had a higher complication rate, a higher complication severity, and most importantly, a higher mortality rate (12% versus 3%). The trial was thus prematurely terminated by the Institutional Review Board. These results were surprising [12,13], given that the trial has been multi-centric and all institutions had a vast experience in pancreatic surgery. In addition, a recent retrospective study published by one of the authors of the trial had demonstrated less complications and comparable mortality with omission of drains after pancreaticoduodenectomy and distal pancreatectomy [8].

Because of these recent publications, we sought to provide pooled estimates of the available literature to allow for a better interpretation of the current evidence.

## Methods

### Literature search

This meta-analysis was performed according to the PRISMA (Preferred Reporting Items for Systematic Reviews and Meta-Analyses) statement [14]. The literature screening was conducted by two independent researchers (U.N. and T.C.M.) in January 2014, without time or language restriction for all articles mentioning the phrases “pancreas” together with “resection” and “drain”. No other limits were applied. Reviewed databases were Pubmed/Medline, Science direct, The Cochrane Central register of controlled trials/Cochrane Library, and EMBASE. Congress abstracts and personal communications were not considered in order to warrant a reproducible meta-analysis of sufficient quality. Articles were included when they reported clinical studies on human subjects with any kind of benign or malignant elective pancreatic resections. The studies needed to compare a group of patients with any kind of pancreatic resection and postoperative intraperitoneal drainage (control group) versus a group of patients with comparable pancreatic resections but no postoperative intraperitoneal drainage (intervention group). Studies reporting on enucleation or drainage for pancreatitis without resection were excluded. We included studies regardless of their design (prospective/retrospective, randomized controlled/non randomized controlled, cohort/case–control) and length of follow up. Identified studies were evaluated by title, abstract, or full reading until inclusion or exclusion criteria were met, respectively. In addition, references of included studies were screened.

### Outcome variables

Eligible studies were evaluated regarding study design, patient population, underlying pancreatic diseases, surgical procedures, and time of recruitment, as reported in Table 1. The primary outcome was overall morbidity after pancreatic resection with or without an intraperitoneal drain. A subgroup analysis was performed for pancreaticoduodenectomy. Secondary outcomes that were not reported in all studies were rates of minor and major complications (according to the Clavien/Dindo classification system [15] grade I-II and III-V, respective), pancreatic fistulas (according to the ISGPF classification system [16] grade B-C), intraabdominal abscesses, the need for interventional radiology procedures (insertion of drainage catheters), the need for re-operations, the length of hospital stay, and mortality (Table 2). To assess study quality and reporting bias, the MINORS [17] and STROBE [18] criteria were applied. Each study was evaluated for the 12 MINORS items with 0, 1, or 2 points, and for all 34 STROBE items with 0 or 1 point, respectively. The results of the MINORS as well as STROBE questions were added and reported in percentages to allow for a review of the individual study quality (Table 1). Zero percent depicts the worst possible study design and 100% depicts a perfectly conducted study. This meta-analysis was registered at the PROSPERO international prospective register of systematic reviews with the number CRD42014007497, as reported on http://www.crd.york.ac.uk/PROSPERO. Because all original data of this study has already been published in individual reports and no kind of research has been performed on living individuals, no ethics approval was requested at the institutional review board.

**Table 1:** Characteristics, patient numbers, and quality of the eight studies that were included in this meta-analysis

**Year**	**Author**	**Study design**	**Patients**	**Inclusion year (from - until)**	**Age (years)**	**Gender (men, women)**	**Disease (malignant, benign)**	**Type of resection (PD, distal, others)**	**Study quality**	
									**MINORS**	**STROBE**
**1998**	**Heslin**[5]	Retrospective	89	1994 - 1996	65 (mean)	50, 39	78, 11	89, 0, 0	83%	58%
**2001**	**Conlon**[6]	Prospective randomized	179	n.s. - n.s.	65 (mean)	89, 90	176, 3	139, 40, 0	79%	77%
**2011**	**Fisher**[8]	Time cohort	226	2004 - 2010	62 (median)	97, 129	113, 113	153, 73, 0	60%	69%
**2012**	**Paulus**[10]	Retrospective	69	1997 - 2011	55 (median)	n.s., n.s.	52, 17	0, 69, 0	65%	55%
**2013**	**Adham**[9]	Retrospective	242	2005 - 2012	62 (median)	127, 115	180, 62	148, 66, 28	58%	66%
**2013**	**Mehta**[11]	Retrospective	709	2005 - 2012	62 (mean)	352, 357	451, 258	709, 0, 0	70%	69%
**2013**	**Correa-Gallego**[7]	Retrospective	1122	2006 - 2011	65 (mean)	548, 574	786, 336	739, 350, 33	50%	60%
**2014**	**Van Buren**[12]	Prospective randomized	137	2011 - 2012	63 (mean)	75, 62	95, 42	137, 0, 0	88%	90%

If not indicated otherwise, numbers of patients are stated. For the explanation of study quality according to MINORS and STROBE criteria, see in the text. n.s., not specified, PD, pancreaticoduodenectomy.

**Table 2:** Numbers of included patients, complication rates, intervention rates, length of hospital stay, and mortality for the drain and no drain group

**Year**	**Author**	**All patients**		**Any complication**		**Minor complication**		**Major complication**		**Fistula (grade B/C)**		**Abscess**		**Rad. Intervention**		**Re-operation**		**Length of hospital stay (d, estimated mean ± SD)**		**Mortality**	
		**Drain**	**No drain**	**Drain**	**No drain**	**Drain**	**No drain**	**Drain**	**No drain**	**Drain**	**No drain**	**Drain**	**No drain**	**Drain**	**No drain**	**Drain**	**No drain**	**Drain**	**No drain**	**Drain**	**No drain**
**1998**	**Heslin**[5]	51	38	23	15	13	9	14	8	3	1	3	0	2	1	1	3	12 ± 7	12 ± 6	n.s.	n.s.
**2001**	**Conlon**[6]	88	91	66	57	93*	83*	43**	39**	11	0	6	6	11	7	8	4	n.s.	n.s.	2	2
**2011**	**Fisher**[8]	179	47	117	22	171***	23***	38	7	21	5	10	2	4	5	8	0	7 ± 2	7 ± 1	1	1
**2012**	**Paulus**[10]	39	30	15	20	n.s.	n.s.	n.s.	n.s.	6	0	8	7	5	7	11	8	9 ± 2.5	6.5 ± 1	1	0
**2013**	**Adham**[9]	130	112	83	45	46	27	32	48	12	13	16	15	8	14	16	17	16.2 ± 24	17.8 ± 31	7	5
**2013**	**Mehta**[11]	251	458	171	248	n.s.	n.s.	62	75	41	35	n.s.	n.s.	21	29	14	26	n.s.	n.s.	5	11
**2013**	**Correa-Gallego**[7]	553	569	301	272	n.s.	n.s.	185	150	149	102	n.s.	n.s.	103****	83*****	3****	2*****	n.s.	n.s.	6****	12*****
**2014**	**Van Buren**[12]	68	69	50	55	n.s.	n.s.	21	28	8	14	8	18	6	16	2	6	7 ± 2	8 ± 5	2	8

These values were used for the meta-analysis. Consider the difficulty of comparing the length of hospital stay due to its varying specifications, as mentioned in the text. d, days, SD, standard deviation, n.s., not specified.

*Out of 176 (multiple mentions).

**Out of 82 (multiple mentions).

***Out of 194 (multiple mentions).

****Out of 540 (subgroup).

*****Out of 549 (subgroup).

### Statistical analysis

The dichotomous data on morbidity and mortality were analyzed in random effects meta-analyses by the Mantel-Haenszel method, using odds ratios (OR) and 95% confidence intervals (CI) as the effect measures. To estimate a pooled mean difference regarding the length of hospital stay using a random effects meta-analysis required the mean and standard deviation of length of stay for each group in each study. If the studies reported on median and interquartile range (Fisher et al. [8], Van Buren et al. [12]) or median and range (Paulus et al. [10], Adham et al. [9]), but not mean and standard deviation, it was estimated as suggested by the Cochrane Handbook for Systematic Reviews of Interventions [19]. Due to the applied approximations, the results regarding length of hospital stay should be interpreted with caution. Inter-study heterogeneity for the respective analyses is shown in the figures as I^2^. Data are displayed in total numbers and are illustrated by Forest plots with p-values and 95% confidence intervals. Publication bias was assessed by a funnel plot. All statistical tests were performed two-sided, and p-values less than 0.05 were considered to be statistically significant. No correction of p-values was applied to adjust for multiple comparisons. However, results of all statistical tests conducted were thoroughly reported, so that an informal adjustment of p-values can be performed while reviewing the data [20]. No additional analyses other than the reported ones were performed. All analyses were performed using the software Revman 5.2.7 for Windows, available online for free at http://ims.cochrane.org/revman/download (downloaded January 04, 2014).

## Results

### Identified studies

**Figure 1:**
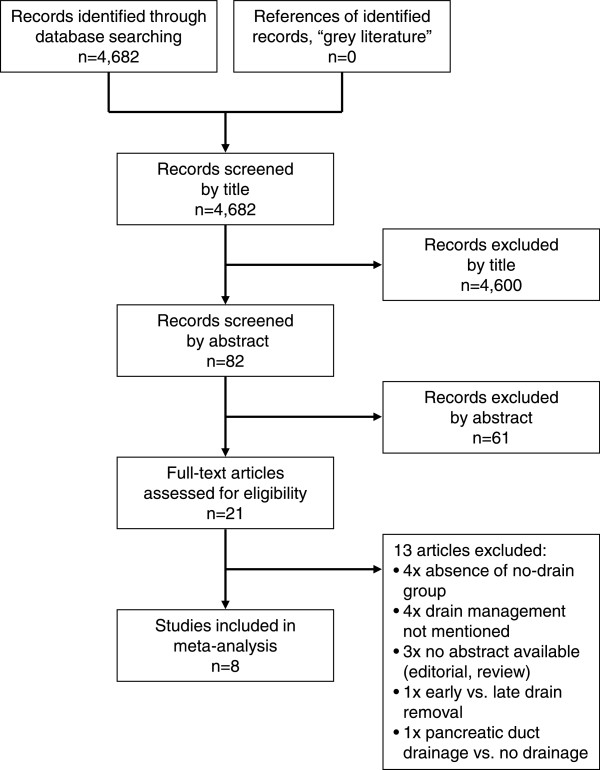
Flow chart with the number of screened, assessed, and finally included studies in the meta-analysis.

Based on the search criteria, 4,682 articles were screened and assessed for eligibility. Of those, duplicates were omitted and 82 abstracts were reviewed in more detail. All abstracts were available either in English or were translated into English. Twenty one studies (all in English) required full reading to decide on inclusion. The remaining 61 studies were excluded because the abstracts revealed that the inclusion criteria were not met. Subsequently, the reference lists of all reviewed articles were checked manually, which did not lead to the identification of additional studies. Figure 1 displays the flow diagram of the screening and inclusion process according to the PRISMA statement [14]. Eight studies met the full inclusion criteria and were included in the meta-analysis. Table 1 gives an overview of the included studies, patient numbers, time spans of recruitment, clinical data, and estimation of the methodological study quality according to MINORS [17] and STROBE [18] criteria. The methodological quality of all studies was at least 50% (of a maximal possible 100%), and assessment regarding MINORS versus STROBE criteria did not show any relevant discrepancies for the individual studies (mean value MINORS: 69%, mean value STROBE: 68%; p = 0.80, paired t test). The smallest study [10] reported on 69 patients, while the largest study [7] reported on 1,122 patients. The two prospective randomized studies were from the years 2001 [6] (n = 179 patients) and 2014 [12] (n = 137 patients). The latter was the only multi-center study [12]; all other studies were single center reports. In total, 1,414 patients without drain were compared to 1,359 patients with drain. Three of all eight studies reported only on pancreaticoduodenectomy and were thus eligible for subgroup analysis [5,11,12]. Additionally, Correa-Gallego et al. [7] reported separately on pancreaticoduodenectomy. Thus, finally four studies were included in the subgroup analysis, comparing 565 patients without drain to 370 patients with drain.

### Drain versus no drain: any complication

**Figure 2:**
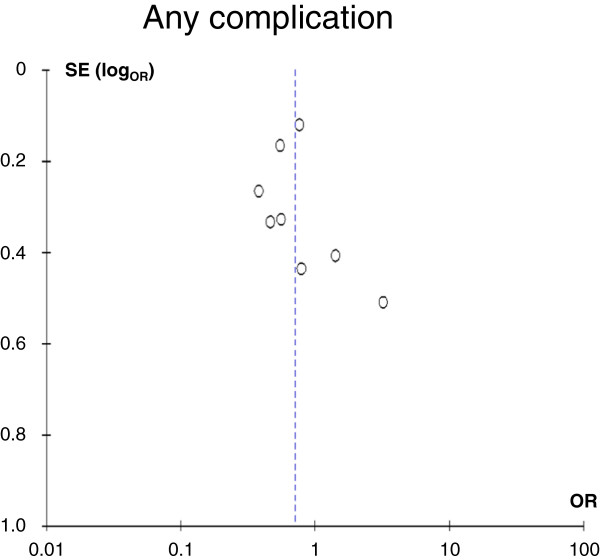
**Funnel plot for the primary outcome “any complication”.** Despite the relatively low number of included studies (n = 8), no clear asymmetry was detected that would suggest selection bias. OR, odds ratio, SE(log_OR_), standard error of the log OR.

**Figure 3:**
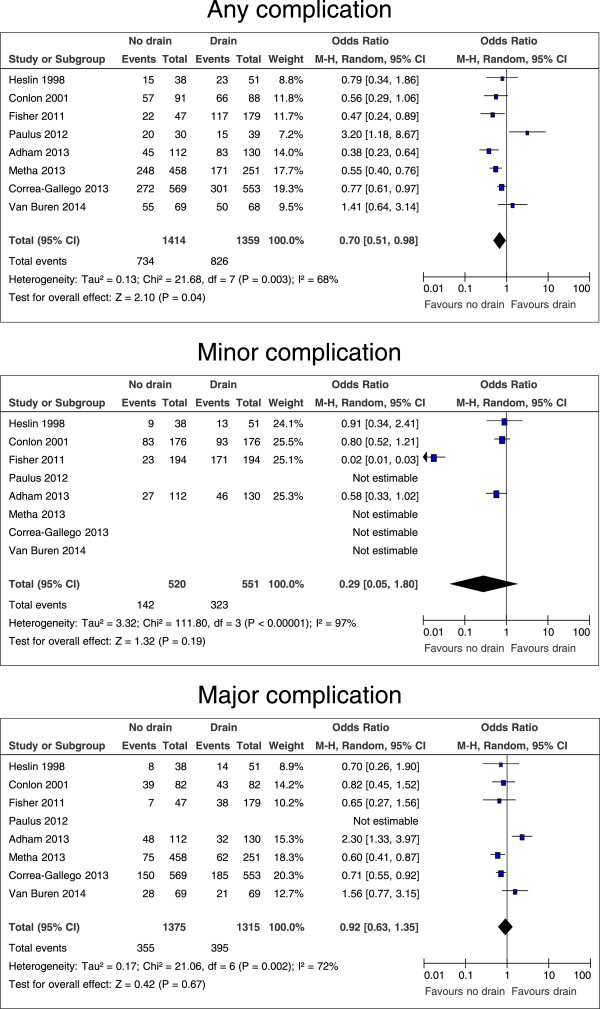
**Forrest plots for risk of any complication, minor and major complications for patients after all kinds of pancreatic resection, with and without intraperitoneal drain.** The overall effect is shown by the diamond, with level of significance (p-value), 95% confidence intervals (CIs), and diversity between studies (Heterogeneity, I^2^).

Table 2 shows the numbers of patients for the parameters that were analyzed. All eight studies and all 2,773 patients were included for the comparison of any complication. A funnel plot for potential publication bias regarding the development of any complication after pancreatic resection with and without drain is shown in Figure 2 and does not provide evidence of publication bias. All studies except for two (including one randomized controlled trial) [10,12] revealed a lower morbidity in the no drain group. Overall heterogeneity was high with I^2^ = 68%. Patients without drain had significantly less complications (p = 0.04, OR 0.70, 95% CI 0.51 – 0.98, Figure 3). If only the two prospective randomized studies [6,12] were taken into account, this effect was not significant (p = 0.75, OR 0.86, 95% CI 0.35 – 2.14, graph not shown).

### Drain versus no drain: specific complications

**Figure 4:**
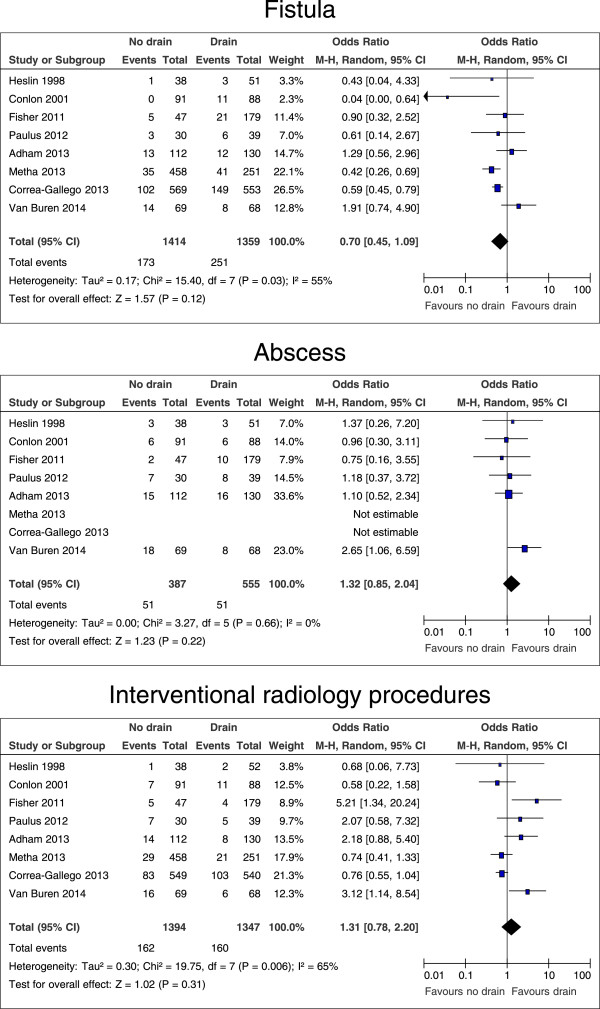
**Forrest plots for risk of fistula, abscess, and interventional radiology procedures for patients after all kinds of pancreatic resection, with and without intraperitoneal drain.** The overall effect is shown by the diamond, with level of significance (p-value), 95% confidence intervals (CIs), and diversity between studies (Heterogeneity, I^2^).

**Figure 5:**
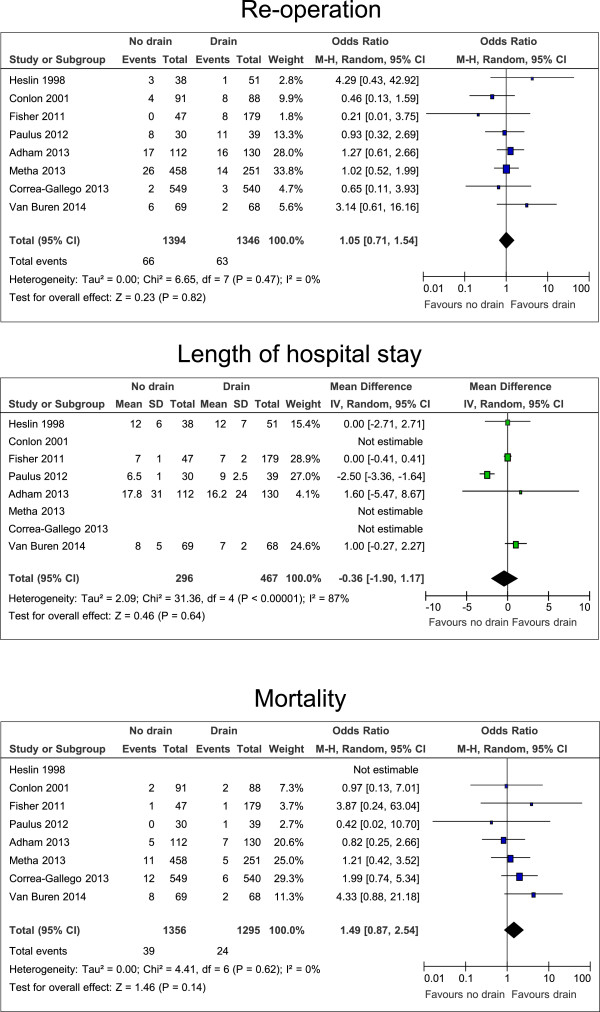
**Forrest plots for risk of re-operation and mortality, and difference in the length of hospital stay for patients after all kinds of pancreatic resection, with and without intraperitoneal drain.** The overall effect is shown by the diamond, with level of significance (p-value), 95% confidence intervals (CIs), and diversity between studies (Heterogeneity, I^2^).

Not all studies reported on the number of patients with minor complications (grade I and II [15]), major complications (grade III – V [15]), pancreatic fistulas (grade B and C [16]), intraabdominal abscesses, the need for interventional radiology procedures, and the need for re-operations. Pooled estimates showed no differences in rates of minor and major complications (p = 0.19 and p = 0.67, respectively), pancreatic fistula, intraabdominal abscess, the numbers of interventional radiology procedures, and re-operations (Figures 3, 4 and 5).

### Drain versus no drain: length of hospital stay and mortality

The length of hospital stay was reported in all eight studies. However, the days of the hospital stay were reported as mean, median, or without any specification, making a robust comparison difficult. Using the estimations as described in the Methods section, there was no significant difference in the length of hospital stay. Postoperative mortality was reported in seven studies, with an observation period of up to three months. Pooled estimates of mortality rates did not show a significant difference (Figure 5).

### Subgroup analysis: pancreaticoduodenectomy

**Figure 6:**
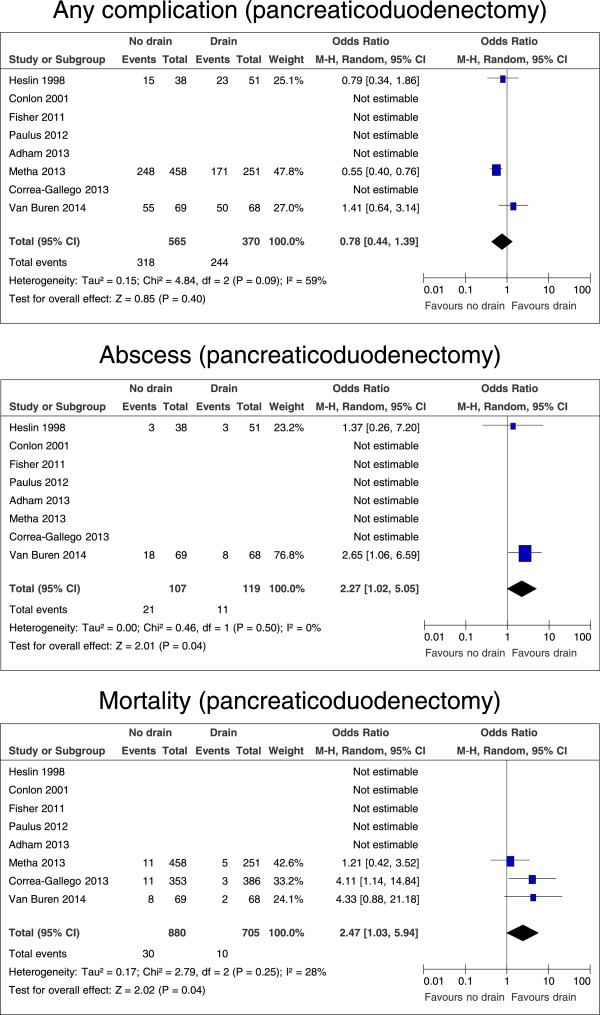
**Forrest plots for risk of any complication, abscess, and mortality for patients after pancreaticoduodenectomy, with and without intraperitoneal drain.** The overall effect is shown by the diamond, with level of significance (p-value), 95% confidence intervals (CIs), and diversity between studies (Heterogeneity, I^2^).

Outcomes after pancreaticoduodenectomy were analyzed separately. Here, no differences were found for any complication (p = 0.40; Figure 6), minor complications (p = 0.85), major complications (p = 0.61), pancreatic fistula (p = 0.63), the need for interventional radiology procedures (p = 0.99), re-operation rates (p = 0.39), or the length of hospital stay (p = 0.16; data not shown). However, without drains the number of intraabdominal abscesses was significantly increased (p = 0.04, OR 2.27, 95% CI 1.02 – 5.05; Figure 6) and mortality was considerably higher (p = 0.04, OR 2.47, 95% CI 1.03 – 5.94; Figure 6).

## Discussion

In this meta-analysis, prophylactic intraperitoneal drainage after pancreatic resection increased the risk of complications, but not the rate of mortality. Pooled estimates of outcomes in the subgroup ‘pancreaticoduodenectomy’ demonstrated different results. Here, the rate of complications was comparable with or without a drain; however, mortality was increased by omission of intraperitoneal drains.

It could be argued that drains following pancreatic resection (as in other abdominal operations) are associated with an, albeit small 1.4-fold (OR 1.43, 95% CI 1.02 – 1.96), increased risk of morbidity without significantly influencing mortality. In the case of pancreaticoduodenectomy, leakage/dehiscence of the pancreatic-intestinal anastomosis is a potentially dreadful complication significantly contributing to the overall mortality following this procedure. From the clinical experience as well as from the data of the included studies, it is possible to conceive that a drain could help to detect a leak earlier and to drain this potentially hazardous fluid (leading to haemorrhage etc.) earlier and more efficiently than an interventional placed drain. Thus, omission of a routine drain following pancreaticoduodenectomy results in a 2.5-fold (OR 2.47, 95% CI 1.03 – 5.94) increased risk of mortality.

These conclusions are supported by the eight included studies, making this meta-analysis the most comprehensive so far. Despite the proper methodological quality of all includes trials, this study has limitations. Because of the different surgical approaches, we performed a subgroup analysis for pancreaticoduodenectomy. In general, there are few studies available investigating the use of drains for pancreatic resections, and only two prospective randomized trials exist. Especially in consideration of the results of the most recent prospective multicenter trial [12], future randomized studies may be ethically difficult to conduct.

The latter trial by Van Buren et al. [12] was stopped preliminary due to the unexpectedly high rate of mortality after pancreaticoduodenectomy in the no drain group. Unfortunately, the reasons for this outcome remain unclear, especially since it was highly contradictory to prior studies. A thorough analysis of the potential factors contributing to these results is beyond the scope of this review. However, it has to be acknowledged that this was the first multicenter study with a well-planned study protocol and the participation of only highly experienced centers. Certainly, the results cannot be explained by the multicentric character, flaws in the study protocol or the lack of experience in the participating centers. As such, the data of the trial by Van Buren et al. have to be taken as high quality evidence.

Recently, a meta-analysis by Rondelli et al. was published, which also meta-analyzed the results of intraperitoneal drainage after pancreatic resection [21]. In contrast to our meta-analysis, the results of the study by Paulus et al. [10] were not included. Comparable to our results, intraperitoneal drainage was identified to be associated with an increase in the total post-operative complication rate (OR 1.52, 95% CI 1.30 – 1.78), and mortality did not differ significantly between the drain/no drain group. Rondelli et al. also performed a subgroup analysis for pancreaticoduodenectomy. As for our analysis, four studies were eligible and the authors found no difference in mortality when analyzing the available randomized study, or the remaining retrospective studies separately. However, when analyzing all studies on pancreaticoduodenectomy together, there was still no significant difference identified in the mentioned meta-analysis, which is in contrast to our results.

## Conclusion

Taking together the data for all pancreatic resections and the subgroup analysis, intraperitoneal drains seem not to be harmful, but may not be beneficial in general either. This would argue for a concept of selective abdominal drain application with placement of a prophylactic drain according to patient factors (e.g. comorbidity, perioperative risk, anticoagulation), pancreatic texture (e.g., small pancreatic duct, soft tissue), surgeon (level of experience, type of operation), and setting (e.g., missing 24/7 availability of interventional radiology procedures) [13]. However, mortality is increased if drains are omitted after pancreaticoduodenectomy. Though this result is driven by the inclusion of the most recent randomized trial into this meta-analysis, it should certainly be taken seriously. Thus, it is very difficult to argue for an omission of drains after a pancreaticoduodenectomy – even under circumstances where anastomotic complications are unlikely. With a reduction of morbidity after any kind of pancreatic resection however, omission of drains after distal pancreatectomy may truly be an option. The results of a currently ongoing randomized trial on distal pancreatectomy are eagerly awaited.

## Competing interests

The authors declare that they have no competing interests.

## Authors’ contributions

UN carried out the review of the literature, participated in performing the meta-analysis and drafted the first version of the manuscript. TCM carried out the independent review of the literature. CS collected data and drafted the manuscript. LC performed and validated the statistical analyses. DW participated in the design of the study and drafted the manuscript. HF interpreted and reviewed the data. CWM made the study concept and drafted the manuscript. JK planned the study, coordinated the drafting of the manuscript, and edited the manuscript. All authors read and approved the final manuscript.

## Pre-publication history

The pre-publication history for this paper can be accessed here:

http://www.biomedcentral.com/1471-2482/14/76/prepub
